# 3-Benzyl-2*H*-chromen-2-one

**DOI:** 10.1107/S1600536812014298

**Published:** 2012-04-21

**Authors:** Guo-Qiang Li, Yao-Lan Li, Tao Jiang, Ren-Wang Jiang, Guo-Cai Wang

**Affiliations:** a Guangdong Province Key Laboratory of Pharmacodynamic Constituents of Traditional Chinese Medicine and New Drugs Research, Institute of Traditional Chinese Medicine and Natural Products, Jinan University, Guangzhou 510632, People’s Republic of China; b Research Center for Harmful Algae and Aquatic Environment, Jinan University, Guangzhou 510632, People’s Republic of China

## Abstract

The title compound, C_16_H_12_O_2_, is a coumarin which was isolated from stones of the Chinese traditional medicine *Clausena lansium*. The pyrone ring is almost planar, with a mean deviation of 0.0135 (4) Å. The benzene ring (*A*) of the benzopyrone unit forms dihedral angles of 1.82 (5) and 72.86 (2)° with the pyrone ring and the substituent benzene ring, respectively. The crystal structure is stabilized by weak π–π stacking inter­actions, with a minimum centroid–centroid distance between benzene rings of 3.6761 (7) Å.

## Related literature

For general background to the isolation of the title compound, see: Wisanu *et al.* (2010 ▶, 2012 ▶). For the biological activity of *Clausena lansium*, see: Adebajo *et al.* (2009 ▶).
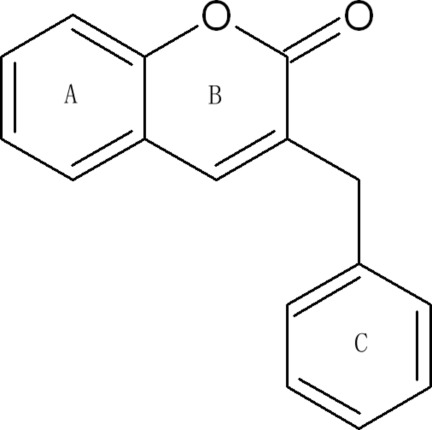


## Experimental

### 

#### Crystal data

C_16_H_12_O_2_
*M* *_r_* = 236.26
Monoclinic, 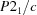

*a* = 11.7704 (4) Å
*b* = 8.2809 (4) Å
*c* = 12.4652 (6) Å
β = 108.151 (2)°
*V* = 1154.52 (9) Å^3^
*Z* = 4
Mo *K*α radiation
μ = 0.09 mm^−1^
*T* = 150 K
0.86 × 0.23 × 0.21 mm

#### Data collection

Bruker SMART CCD 1000 diffractometer
Absorption correction: multi-scan (*SADABS*; Sheldrick, 1996 ▶) *T*_min_ = 0.928, *T*_max_ = 0.982
8002 measured reflections
2475 independent reflections
2050 reflections with *I* > 2σ(*I*)
*R*_int_ = 0.033

#### Refinement

*R*[*F* ^2^ > 2σ(*F* ^2^)] = 0.034
*wR*(*F* ^2^) = 0.093
*S* = 1.08
2475 reflections
163 parameters
H-atom parameters constrained
Δρ_max_ = 0.24 e Å^−3^
Δρ_min_ = −0.18 e Å^−3^

### 

Data collection: *SAINT* (Bruker, 1998 ▶); cell refinement: *SMART* (Bruker, 1998 ▶); data reduction: *SAINT*; program(s) used to solve structure: *SHELXS97* (Sheldrick, 2008 ▶); program(s) used to refine structure: *SHELXL97* (Sheldrick, 2008 ▶); molecular graphics: *OLEX2* (Dolomanov *et al.*, 2009 ▶); software used to prepare material for publication: *OLEX2*.

## Supplementary Material

Crystal structure: contains datablock(s) I, global. DOI: 10.1107/S1600536812014298/zs2199sup1.cif

Structure factors: contains datablock(s) I. DOI: 10.1107/S1600536812014298/zs2199Isup2.hkl

Supplementary material file. DOI: 10.1107/S1600536812014298/zs2199Isup3.cml

Additional supplementary materials:  crystallographic information; 3D view; checkCIF report

## Figures and Tables

**Fig. 1.**
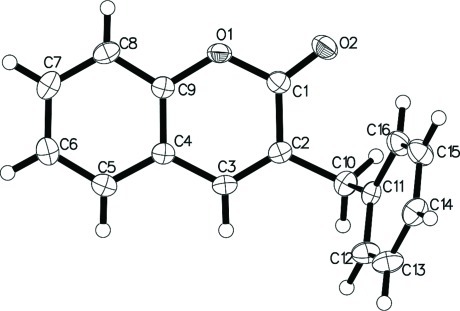
The molecular structure of the title compound showing 50% probability displacement ellipsoids and the atom-numbering scheme.

**Fig. 2.**
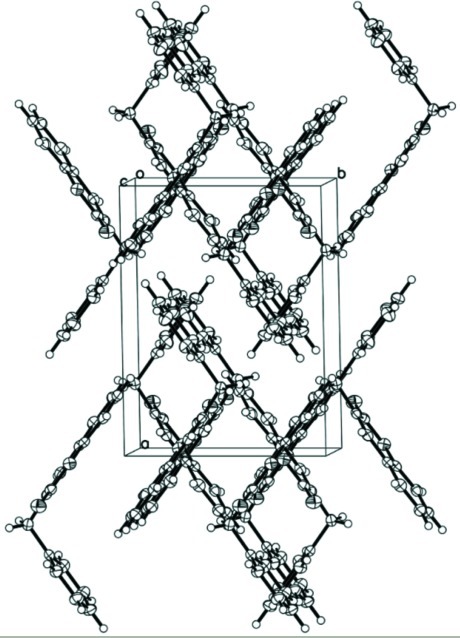
The packing of the title compound, viewed down the c axis.

## supplementary crystallographic information

### Comment

The title compound, C_16_H_12_O_2_ (systematic name:3-benzylchromen-2-one), is a coumarin which was isolated from stones of the Chinese traditional medicine *Clausena lansium*. This plant is a rich source of coumarin (Wisanu *et al.*, 2010; Wisanu *et al.*, 2012). The biological activity of *Clausena lansium* have been studied (Adebajo *et al.*, 2009). In this study, we report the crystal structure of the title compound (Fig. 1) comprises two benzene rings (*A* and *C*) and a pyrone ring (*B*), which is almost planar with a mean deviation 0.0135 (4) Å. The ring *A* of the benzopyrone unit forms dihedral angles of 1.82 (5) and 72.86 (2)° with the ring *B* and the ring *C*, respectively. The molecules are stacked parallel to the *c* axis giving weak π–π interactions between benzene rings (Fig. 2), with a minimum centroid–centroid distance of 3.6761 (7) Å.

### Experimental

The title compound was isolated from stones of the traditional chinese medicine Clausena lansium, 5 kg of which was extracted with 95% ethanol at room temperature, then concentrated by rotary evaporation. The crude extract was suspended in distilled water and partitioned with petroleum ether, ethyl acetate and n-butanol. The title compound (8 mg) was isolated from the petroleum ether fraction using silica gel column chromatography. Crystals of the title compound were obtained after slow evaporation of an ethyl acetate solution at room temperature.

### Refinement

All H atoms were positioned geometrically and were included in the refinement in the riding-model approximation, with C—H = 0.99 Å (CH_2_) or C—H = 0.95 Å (aryl H) and *U*_iso_(H)= 1.2*U*_eq_(C).

### Crystal data

**Table:**

C_16_H_12_O_2_	*F*(000) = 496
*M**_r_* = 236.26	*D*_x_ = 1.359 Mg m^−^^3^
Monoclinic, *P*2_1_/*c*	Mo *K*α radiation, λ = 0.710747 Å
*a* = 11.7704 (4) Å	Cell parameters from 8002 reflections
*b* = 8.2809 (4) Å	θ = 3–27.5°
*c* = 12.4652 (6) Å	µ = 0.09 mm^−^^1^
β = 108.151 (2)°	*T* = 150 K
*V* = 1154.52 (9) Å^3^	Prism, colourless
*Z* = 4	0.86 × 0.23 × 0.21 mm

### Data collection

**Table:**

Bruker SMART CCD 1000 diffractometer	2475 independent reflections
Radiation source: fine-focus sealed tube	2050 reflections with *I* > 2σ(*I*)
Graphite monochromator	*R*_int_ = 0.033
ω scans	θ_max_ = 27.0°, θ_min_ = 3.0°
Absorption correction: multi-scan (*SADABS*; Sheldrick, 1996)	*h* = −14→13
*T*_min_ = 0.928, *T*_max_ = 0.982	*k* = −10→10
8002 measured reflections	*l* = −12→15

### Refinement

**Table:**

Refinement on *F*^2^	Primary atom site location: structure-invariant direct methods
Least-squares matrix: full	Secondary atom site location: difference Fourier map
*R*[*F*^2^ > 2σ(*F*^2^)] = 0.034	Hydrogen site location: inferred from neighbouring sites
*wR*(*F*^2^) = 0.093	H-atom parameters constrained
*S* = 1.08	*w* = 1/[σ^2^(*F*_o_^2^) + (0.0426*P*)^2^ + 0.2137*P*] where *P* = (*F*_o_^2^ + 2*F*_c_^2^)/3
2475 reflections	(Δ/σ)_max_ < 0.001
163 parameters	Δρ_max_ = 0.24 e Å^−^^3^
0 restraints	Δρ_min_ = −0.18 e Å^−^^3^

### Special details

**Table:**

Geometry. All e.s.d.'s (except the e.s.d. in the dihedral angle between two l.s. planes) are estimated using the full covariance matrix. The cell e.s.d.'s are taken into account individually in the estimation of e.s.d.'s in distances, angles and torsion angles; correlations between e.s.d.'s in cell parameters are only used when they are defined by crystal symmetry. An approximate (isotropic) treatment of cell e.s.d.'s is used for estimating e.s.d.'s involving l.s. planes.
Refinement. Refinement of *F*^2^ against ALL reflections. The weighted *R*-factor *wR* and goodness of fit *S* are based on *F*^2^, conventional *R*-factors *R* are based on *F*, with *F* set to zero for negative *F*^2^. The threshold expression of *F*^2^ > σ(*F*^2^) is used only for calculating *R*-factors(gt) *etc*. and is not relevant to the choice of reflections for refinement. *R*-factors based on *F*^2^ are statistically about twice as large as those based on *F*, and *R*- factors based on ALL data will be even larger.

### Fractional atomic coordinates and isotropic or equivalent isotropic displacement parameters (Å^2^)

**Table:**

	*x*	*y*	*z*	*U*_iso_*/*U*_eq_	
C1	0.38027 (9)	0.35804 (12)	0.36947 (9)	0.0229 (2)	
C2	0.35856 (9)	0.38688 (11)	0.47770 (9)	0.0206 (2)	
C3	0.43498 (9)	0.32578 (12)	0.57355 (9)	0.0213 (2)	
H3	0.4219	0.3477	0.6436	0.026*	
C4	0.53617 (9)	0.22788 (12)	0.57215 (9)	0.0206 (2)	
C5	0.61806 (10)	0.16006 (13)	0.66886 (10)	0.0260 (3)	
H5	0.6099	0.1803	0.7411	0.031*	
C6	0.71066 (10)	0.06388 (13)	0.65934 (10)	0.0287 (3)	
H6	0.7660	0.0191	0.7252	0.034*	
C7	0.72314 (10)	0.03238 (12)	0.55376 (11)	0.0271 (3)	
H7	0.7868	−0.0342	0.5482	0.033*	
C8	0.64344 (9)	0.09730 (12)	0.45677 (10)	0.0251 (2)	
H8	0.6511	0.0752	0.3846	0.030*	
C9	0.55206 (9)	0.19552 (12)	0.46798 (9)	0.0206 (2)	
C10	0.24760 (9)	0.48264 (12)	0.47378 (10)	0.0244 (2)	
H10A	0.2366	0.5706	0.4177	0.029*	
H10B	0.2586	0.5325	0.5485	0.029*	
C11	0.13605 (9)	0.37716 (12)	0.44253 (9)	0.0216 (2)	
C12	0.09172 (10)	0.31780 (13)	0.52614 (10)	0.0285 (3)	
H12	0.1305	0.3453	0.6029	0.034*	
C13	−0.00877 (11)	0.21854 (14)	0.49858 (11)	0.0323 (3)	
H13	−0.0383	0.1794	0.5565	0.039*	
C14	−0.06579 (10)	0.17677 (13)	0.38743 (11)	0.0296 (3)	
H14	−0.1344	0.1092	0.3687	0.036*	
C15	−0.02205 (10)	0.23422 (14)	0.30374 (11)	0.0307 (3)	
H15	−0.0604	0.2051	0.2273	0.037*	
C16	0.07789 (9)	0.33453 (13)	0.33096 (10)	0.0272 (3)	
H16	0.1066	0.3742	0.2727	0.033*	
O1	0.47592 (6)	0.26132 (9)	0.36983 (6)	0.02389 (19)	
O2	0.32169 (7)	0.41253 (10)	0.27881 (7)	0.0331 (2)	

### Atomic displacement parameters (Å^2^)

**Table:**

	*U*^11^	*U*^22^	*U*^33^	*U*^12^	*U*^13^	*U*^23^
C1	0.0211 (5)	0.0238 (5)	0.0243 (6)	−0.0041 (4)	0.0078 (4)	0.0014 (4)
C2	0.0205 (5)	0.0186 (5)	0.0242 (6)	−0.0052 (4)	0.0092 (4)	−0.0015 (4)
C3	0.0233 (5)	0.0221 (5)	0.0209 (6)	−0.0049 (4)	0.0102 (4)	−0.0034 (4)
C4	0.0204 (5)	0.0199 (5)	0.0221 (6)	−0.0050 (4)	0.0076 (4)	−0.0012 (4)
C5	0.0273 (6)	0.0286 (5)	0.0217 (6)	−0.0024 (4)	0.0072 (4)	−0.0003 (4)
C6	0.0246 (6)	0.0268 (5)	0.0318 (7)	−0.0005 (4)	0.0045 (5)	0.0045 (5)
C7	0.0231 (5)	0.0199 (5)	0.0401 (7)	−0.0018 (4)	0.0126 (5)	−0.0016 (4)
C8	0.0262 (6)	0.0237 (5)	0.0294 (6)	−0.0053 (4)	0.0143 (5)	−0.0050 (4)
C9	0.0201 (5)	0.0199 (5)	0.0221 (6)	−0.0053 (4)	0.0071 (4)	−0.0006 (4)
C10	0.0238 (6)	0.0203 (5)	0.0292 (6)	−0.0012 (4)	0.0086 (4)	−0.0014 (4)
C11	0.0198 (5)	0.0179 (5)	0.0276 (6)	0.0031 (4)	0.0079 (4)	0.0001 (4)
C12	0.0312 (6)	0.0310 (6)	0.0254 (6)	−0.0042 (5)	0.0121 (5)	−0.0067 (5)
C13	0.0358 (6)	0.0343 (6)	0.0338 (7)	−0.0070 (5)	0.0207 (5)	−0.0034 (5)
C14	0.0224 (6)	0.0297 (6)	0.0372 (7)	−0.0056 (4)	0.0101 (5)	−0.0021 (5)
C15	0.0264 (6)	0.0368 (6)	0.0247 (6)	−0.0043 (5)	0.0020 (5)	0.0011 (5)
C16	0.0253 (6)	0.0307 (6)	0.0250 (6)	−0.0020 (4)	0.0067 (4)	0.0066 (4)
O1	0.0247 (4)	0.0297 (4)	0.0191 (4)	−0.0007 (3)	0.0094 (3)	0.0003 (3)
O2	0.0314 (4)	0.0432 (5)	0.0240 (5)	0.0019 (4)	0.0077 (3)	0.0089 (4)

### Geometric parameters (Å, º)

**Table:**

C1—C2	1.4687 (15)	C8—C9	1.3898 (15)
C1—O1	1.3805 (13)	C9—O1	1.3834 (13)
C1—O2	1.2128 (13)	C10—H10A	0.9900
C2—C3	1.3502 (15)	C10—H10B	0.9900
C2—C10	1.5157 (14)	C10—C11	1.5231 (14)
C3—H3	0.9500	C11—C12	1.3925 (15)
C3—C4	1.4453 (14)	C11—C16	1.3908 (16)
C4—C5	1.4050 (15)	C12—H12	0.9500
C4—C9	1.3944 (16)	C12—C13	1.3927 (16)
C5—H5	0.9500	C13—H13	0.9500
C5—C6	1.3845 (16)	C13—C14	1.3821 (18)
C6—H6	0.9500	C14—H14	0.9500
C6—C7	1.3937 (17)	C14—C15	1.3836 (17)
C7—H7	0.9500	C15—H15	0.9500
C7—C8	1.3871 (16)	C15—C16	1.3930 (15)
C8—H8	0.9500	C16—H16	0.9500
			
O1—C1—C2	117.80 (9)	O1—C9—C8	116.75 (10)
O2—C1—C2	125.76 (10)	C2—C10—H10A	109.2
O2—C1—O1	116.43 (10)	C2—C10—H10B	109.2
C1—C2—C10	116.75 (9)	C2—C10—C11	111.95 (8)
C3—C2—C1	119.56 (9)	H10A—C10—H10B	107.9
C3—C2—C10	123.67 (10)	C11—C10—H10A	109.2
C2—C3—H3	119.2	C11—C10—H10B	109.2
C2—C3—C4	121.59 (10)	C12—C11—C10	120.34 (10)
C4—C3—H3	119.2	C16—C11—C10	121.18 (10)
C5—C4—C3	124.16 (10)	C16—C11—C12	118.46 (10)
C9—C4—C3	117.98 (10)	C11—C12—H12	119.6
C9—C4—C5	117.84 (10)	C11—C12—C13	120.74 (11)
C4—C5—H5	119.9	C13—C12—H12	119.6
C6—C5—C4	120.29 (11)	C12—C13—H13	119.8
C6—C5—H5	119.9	C14—C13—C12	120.33 (11)
C5—C6—H6	119.8	C14—C13—H13	119.8
C5—C6—C7	120.40 (11)	C13—C14—H14	120.3
C7—C6—H6	119.8	C13—C14—C15	119.42 (11)
C6—C7—H7	119.7	C15—C14—H14	120.3
C8—C7—C6	120.58 (10)	C14—C15—H15	119.8
C8—C7—H7	119.7	C14—C15—C16	120.38 (11)
C7—C8—H8	120.9	C16—C15—H15	119.8
C7—C8—C9	118.28 (11)	C11—C16—C15	120.67 (11)
C9—C8—H8	120.9	C11—C16—H16	119.7
C8—C9—C4	122.59 (10)	C15—C16—H16	119.7
O1—C9—C4	120.66 (9)	C1—O1—C9	122.30 (9)
			
C1—C2—C3—C4	−1.97 (14)	C7—C8—C9—O1	178.51 (9)
C1—C2—C10—C11	82.11 (11)	C8—C9—O1—C1	178.61 (9)
C2—C1—O1—C9	−1.63 (13)	C9—C4—C5—C6	−0.30 (15)
C2—C3—C4—C5	−179.48 (9)	C10—C2—C3—C4	176.63 (9)
C2—C3—C4—C9	−1.02 (14)	C10—C11—C12—C13	−178.67 (10)
C2—C10—C11—C12	98.80 (12)	C10—C11—C16—C15	178.15 (10)
C2—C10—C11—C16	−79.60 (12)	C11—C12—C13—C14	0.32 (18)
C3—C2—C10—C11	−96.53 (12)	C12—C11—C16—C15	−0.27 (16)
C3—C4—C5—C6	178.17 (9)	C12—C13—C14—C15	0.10 (18)
C3—C4—C9—C8	−177.27 (9)	C13—C14—C15—C16	−0.60 (18)
C3—C4—C9—O1	2.74 (14)	C14—C15—C16—C11	0.70 (17)
C4—C5—C6—C7	−0.46 (16)	C16—C11—C12—C13	−0.23 (16)
C4—C9—O1—C1	−1.39 (14)	O1—C1—C2—C3	3.29 (14)
C5—C4—C9—C8	1.30 (15)	O1—C1—C2—C10	−175.41 (8)
C5—C4—C9—O1	−178.70 (8)	O2—C1—C2—C3	−176.32 (10)
C5—C6—C7—C8	0.27 (16)	O2—C1—C2—C10	4.98 (15)
C6—C7—C8—C9	0.68 (15)	O2—C1—O1—C9	178.02 (9)
C7—C8—C9—C4	−1.49 (15)		
